# Electrophysiological Properties of CA1 Pyramidal Neurons along the Longitudinal Axis of the Mouse Hippocampus

**DOI:** 10.1038/srep38242

**Published:** 2016-12-06

**Authors:** Giampaolo Milior, Maria Amalia Di Castro, Livio Pepe’ Sciarria, Stefano Garofalo, Igor Branchi, Davide Ragozzino, Cristina Limatola, Laura Maggi

**Affiliations:** 1 Pasteur Institute Rome-Department of Physiology and Pharmacology, Sapienza University of Rome, Italy; 2 Inserm U1127, CNRS UMR7225, Sorbonne Universités, UPMC UMR S1127, Institut du Cerveau et de la Moelle épinière, Paris 75013, France; 3 Section of Behavioural Neurosciences, Department of Cell Biology and Neurosciences, Istituto Superiore di Sanità, Rome, Italy; 4 IRCCS Neuromed, Pozzilli, IS, Italy

## Abstract

Evidence for different physiological properties along the hippocampal longitudinal axis is emerging. Here, we examined the electrophysiological features of neurons at different dorso-ventral sites of the mouse CA1 hippocampal region. Cell position was defined with respect to longitudinal coordinates of each slice. We measured variations in neuronal excitability, subthreshold membrane properties and neurotransmitter responses along the longitudinal axis. We found that (i) pyramidal cells of the dorsal hippocampus (DH) were less excitable than those of the ventral hippocampus (VH). Resting Membrane Potential (RMP) was more hyperpolarized and somatic Input Resistance (Ri) was lower in DH compared to VH. (ii) The Paired-pulse ratio (PPR) of focally induced synaptic responses was systematically reduced from the DH to the VH; (iii) Long-term-potentiation was most pronounced in the DH and fell gradually in the intermediate hippocampus and in the VH; (iv) the frequency of miniature GABAergic events was higher in the VH than in the DH; (v) the PPR of evoked inhibitory post-synaptic current (IPSC) was higher in the DH than in the VH. These findings indicate an increased probability of both GABA and glutamate release and a reduced plasticity in the ventral compared to more dorsal regions of the hippocampus.

The hippocampus is a medial temporal lobe structure widely studied for its unique role in learning and memory. In the rodent brain, the hippocampus is an elongated structure with a longitudinal axis extending in a C-shaped fashion from dorsal (septal)-to-ventral (temporal), corresponding to a posterior-to-anterior axis in human^1^. While intrinsic circuitry is conserved along the longitudinal axis, dorsal and ventral regions have different connectivity with cortical and subcortical areas. The dorsal hippocampus receives visual and spatial information from sensory cortices via the medial entorhinal cortex. In contrast, ventral hippocampus is connected to the amygdala, prefrontal cortex (PFC) and hypothalamus^2^,^3^,^4^. Previous studies based on targeted lesions, electrophysiological recording and selective pharmacological blockade suggest that dorsal regions serve mainly cognitive functions, including spatial and declarative memory, while ventral regions are involved in regulating emotional responses^5^,^6^.

Several studies based on gene expression, anatomical and behavioral measurements have suggested that the hippocampus has different functional organizations along its longitudinal axis. In particular, it has been proposed that either this brain region is organized in multiple functional domains or it is structured according to a spatial gradient^2^,^5^,^7^,^8^,^9^. Recently, it has been proposed a model in which functional long-axis gradients are superimposed on tripartite functional domains: ventral (VH), intermediate (IH) and dorsal (DH) regions^1^.

Longitudinal variations in the electrophysiology of hippocampal neurons have been already investigated in the rat. CA1 pyramidal cells have been reported to be more excitable in the ventral compared to the dorsal region^10^,^11^. In addition, the short- and long-term plasticity of synapses terminating on DH and VH neurons differs both *in vivo*^12^ and *in vitro*^13^,^14^,^15^,^16^. Specifically, paired pulse facilitation (PPF), a short-term synaptic plasticity depending on changes in transmitter release^17^, is weaker in VH than in DH^12^,^15^, suggesting that basal transmitter release probability may be higher in VH. Furthermore, the magnitude of long-term potentiation (LTP)^18^ elicited in the VH is significantly smaller than that induced in both DH^14^,^16^ and IH^19^. Though these results provide an electrophysiological characterization along the hippocampal longitudinal axis in the rat, only very limited amount of data is available for the mouse^20^, prompting the need to extend the study also to this species.

In the present study, we investigated in the mouse the electrophysiological properties of CA1 pyramidal cells along the hippocampal longitudinal axis. Neuronal properties, including subthreshold membrane behavior and firing activity, as well as short- and long-term plasticity at synapses that excites these cells were assessed. Furthermore, we examined for the first time the inhibitory GABAergic synaptic function in DH and VH. To this purpose, we exploited an experimental strategy, based on isolating and straightening the hippocampus, to determine the coordinates of the neurons along the dorsal to ventral axis.

## Results

**Figure 1:**
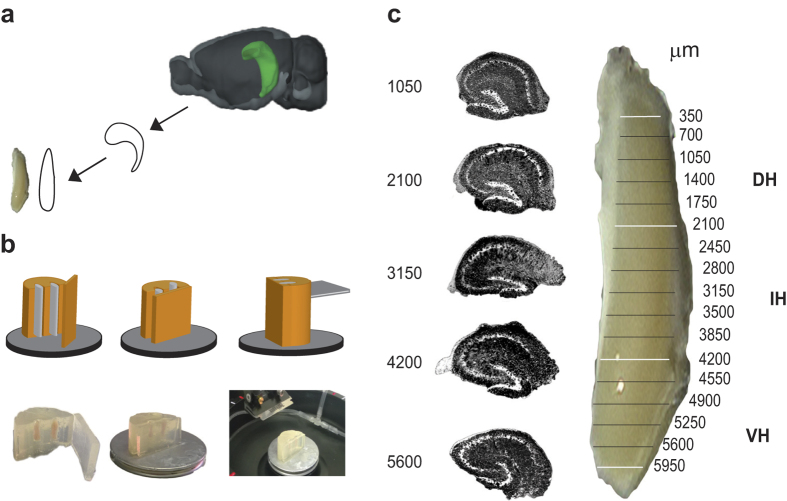
Hippocampal slicing procedure. (**a**) Schematic diagram showing isolation and elongation of the hippocampus. Image credit: © 2015 Allen Institute for Brain Science. Allen Brain Atlas API. (**b**) The hippocampus is placed in a small agarose-block fixed to the cutting stage of the vibratome magnetic plate in order to prepare 350 μm thick transverse slices. (**c**) Schematic partition of the hippocampus along the septo-temporal axis and representative Hoechst- stained slices at different septo-temporal positions distances. White lines indicate boundaries among different zones, the black ones indicate the longitudinal position of slices used for electrophysiological recording.

The procedure used to collect and straighten the hippocampus is described in Fig. 1a,b. This procedure allowed to identify the septo-temporal coordinates of each slice where CA1 pyramidal cells activity was recorded. After the straightening procedure, the average length of the hippocampus was 6.5 ± 0.06 mm (n = 41). The longitudinal coordinates of the DH, IH and VH are presented in Fig. 1c together with representative Hoechst-stained slices at 5 different levels. We analyzed the mid-CA1 region of transverse slices from the DH, IH and VH regions taken in random order to minimize possible effects of time after slicing on electrophysiological parameters.

### Basal responses and excitability of CA1 neurons

#### Input/output curves

**Figure 2:**
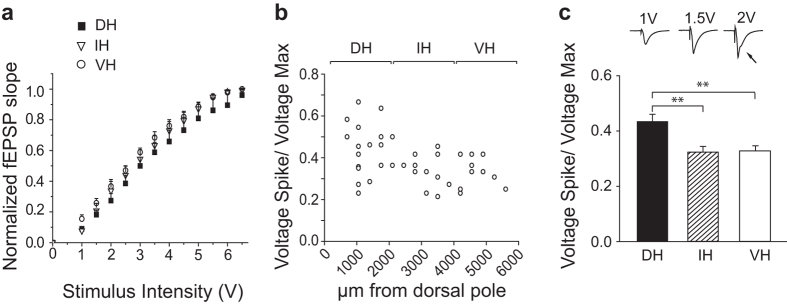
Extracellular fields reveal excitability variation along the septo-temporal axis. Local field potentials induced in the CA1 stratum radiatum by stimulating Schaffer collaterals. (**a**) Curves of normalized fEPSP slope plotted against stimulus intensity (I–O) for records from DH (n = 19/14), IH (n = 12/10) and VH (n = 12/8). (**b**) Current intensity for population spike threshold plotted against distance (μm) from the hippocampal dorsal pole. (**c**) Mean and S.E.M of the spike voltage normalized to the plateau voltage for events induced in DH (0.43 ± 0.03, n = 19/14), IH (0.32 ± 0.02, n = 12/9) and VH (0.33 ± 0.02, n = 14/10). Top: example of field events induced by increasing stimuli in one slice with an arrow showing the population spike at threshold. **Indicates p < 0.01.

As a first step, we recorded field potentials induced in the CA1 stratum pyramidale in response to stimulation of Schaffer collaterals in the same region. Figure 2a shows I-O curves constructed by measuring the slope of fEPSPs elicited by stimuli of graded intensities. Neither the threshold, the peak or the overall curves were markedly different for slices prepared from the DH, IH or the VH regions of the hippocampus. These results are in line with the *in vitro* and *in vivo* data concerning the rat^12^,^15^,^16^.

#### Intrinsic excitability and sub-threshold membrane properties

Synaptically induced action potentials are observed in field potentials as positive-going population spike deflections in records from the stratum radiatum (Fig. 2c, *top right,* black arrow). The minimal stimulation intensity at which they are generated provides a global measure of the excitability of the CA1 pyramidal cell population^21^. As an index of population excitability, we calculated a ratio consisting of the minimal stimulation intensity (V) which evoked a population spike divided by the stimulation intensity which induces fEPSPs of maximal initial slope. Figure 2b,c show that this ratio was higher in slices from DH than for those taken from IH or VH (F = 7.280, p = 0.002; Post-hoc comparisons: DH vs. IH, t = 3.172; p = 0.006; DH vs. VH, t = 3.197, p = 0.008). Thus CA1 pyramidal cells from DH seem to be less excitable than those of IH and VH.

**Figure 3:**
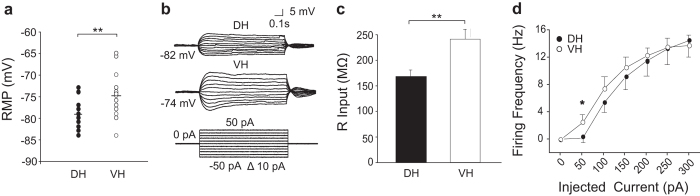
Subthreshold responses and excitability of DH and VH neurons. Whole cell recordings from CA1 pyramidal neurons. (**a**) Resting membrane potential (RMP) was significantly more depolarized for VH (white circles, average: −74.94 ± 1.03 mV, n = 15/8/8) than DH neurons (black circles, average: −79.34 ± 0.81 mV, n = 19/9/9). (**b)** Voltage responses from DH (upper) and VH (lower) pyramidal cells at RMP to 800 ms step current injections ranging from −50 pA to 50 pA in 10 pA increments. (**c**) Rin at RMP was significantly higher for VH (white bar, Rin VH = 235.44 ± 19.43 MΩ, n = 18/11/7) than DH cells (black bar, Rin DH = 167.68 ± 15.8 MΩ, n = 14/10/10). (**d**) Relations between injected current and firing frequency (F–I) for DH (n = 15/9/9) and VH (n = 15/7/7) pyramidal cells. Differences between F-I curves were statistically significant at 50 pA. *Indicates p < 0.05. **Indicates p < 0.01.

In order to explore the cellular basis for this difference, we made whole-cell recordings from CA1 neurons in slices prepared from different septo-temporal sites of the hippocampus. Resting membrane potential (RMP), input resistance (Rin) and neuronal firing properties might all contribute to differences in excitability. In our experimental conditions, CA1 pyramidal cells did not discharge spontaneously. Their RMP was measured as the mean membrane potential maintained over 5–10 min with no injected current. Figure 3a shows that the RMP of VH neurons was more depolarized than that for pyramidal cells from DH regions (t = −3.216, p = 0.002). In addition, the somatic values of Rin were higher for VH neurons than for DH ones (Fig. 3b, t = 2.719, p = 0.010; Fig. 3b) and cell capacitance was not significantly different (DH: 147.22 ± 13.29 pF; 16/10/10; VH: 171.34 ± 9.43 pF, 18/11/7; p = 0.14). To better address whether the differences observed in Rin could depend on the size of the neurons we calculated the specific conductance for each cell as the ratio between conductance and capacitance. We found that these values were similar in DH and VH neurons (DH: 0.055 ± 0.005 nS/μF; 16/10/10; VH: 0.046 ± 0.004, 16/10/7; p = 0.18, nS/μF), suggesting a possible contribution of the soma and primary dendrites size in the Rin diversity. Further data on differences in excitability of CA1 pyramidal cells from DH and VH regions was obtained from responses to depolarizing current steps injected to induce firing. We analyzed curves of discharge frequency, F, plotted against somatically injected current. Figure 3d shows that VH neurons fire action potentials in response to significantly smaller current injections starting at RMP than their DH counterpart (at 50 pA, p = 0.045), indicating that VH cells have lower rheobase, similarly to those reported in rats^10^,^11^.

These results overall confirm that ventral cells are more excitable than pyramidal cells from dorsal hippocampus due to a more depolarized membrane potential and a higher input resistance.

### Short and long term synaptic plasticity along hippocampal septo-temporal axis

**Figure 4:**
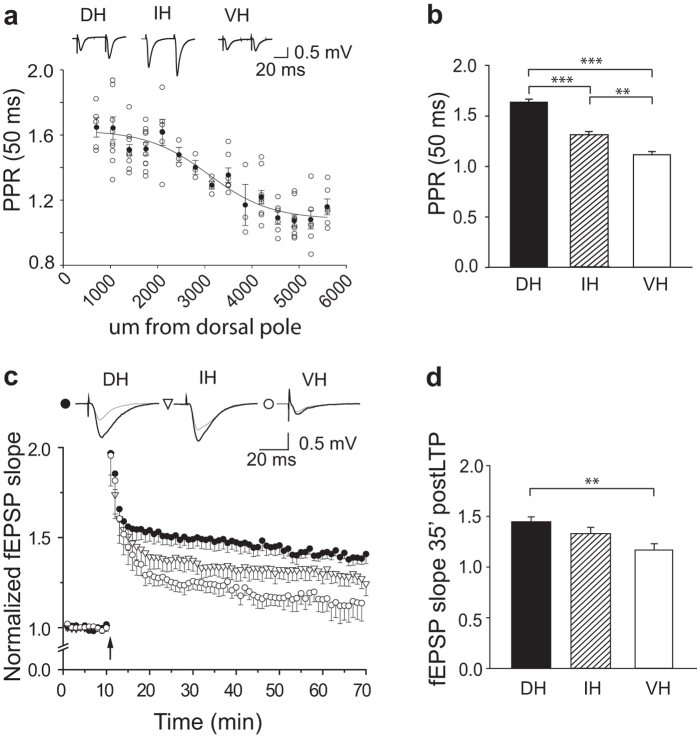
Short and long term plasticity at Schaffer collateral synapses in DH, IH and VH. (**a**) PPR values for fEPSP slope plotted against distance from the dorsal pole (μm) measured from field records at different distances along the septo-temporal hippocampal axis. White circles indicate absolute values of PPR along the septo-temporal axis, and black circles show mean PPR values. The dependence of PPR from slice depth was described assuming a Boltzmann function (f = y0+a/(1+exp (-(x-x0)/b)) with the following fit parameters: dorsal pole distance for 1/2 maximal PPR (x0): 3087.53 ± 240.41 μm, y0 = 1.0838 ± 0.0536, a = 0.5468 ± 0.0950, b = −654.3618 ± 253.5688; n = 15). Note that the interpolating sigmoid function was not very far from a linear interpolating one. Above, fEPSP traces show variation in PPR for slices from DH, IH and VH zones. (**b**) Averaged PPR values for DH was 1.64 ± 0.03 (n = 37/21) in IH slices it was 1.31 ± 0.03 (n = 17/12) and in slices from the VH the PPR was 1.12 ± 0.03 (n = 19/11). (**c)** LTP of fEPSP slope from extracellular records made from the DH (n = 11/11, black circles), IH (n = 7/6, white triangle) and VH (n = 10/9, white circles). Time course of slope values from responses evoked at 0.05 Hz and normalized as detailed in the Methods. Arrows indicate time of HFS (2 trains at 100 Hz of 1 s duration with 3 s inter-train interval). Above are representative traces taken before (gray) and 35 minutes (black) after HFS for each septo-temporal region. (**d**) LTP of fEPSP slope measured at 35 minutes after HFS from slices of the DH, IH and VH. The mean increase was 1.48 ± 0.05 for DH (n = 10/10), 1.33 ± 0.06 for IH (n = 5/5) and 1.19 ± 0.06 for VH (n = 8/7). Data in B and D are mean ± S.E.M. *Indicates p < 0.05 and **indicates p < 0.01. All field waveforms are averaged from three traces.

If intrinsic neuronal properties vary along the septo-temporal axis, do synaptic properties also change? Figure 2a shows that the peak amplitudes of fEPSPs were similar in DH, IH and VH. We asked if short or long-term plasticity of Schaffer collateral synapses made with CA1 pyramidal cells were constant or variable. The paired pulse ratio (PPR) is a form of short-term plasticity depending on neurotransmitter release probability^22^. We determined the PPR of fEPSPs induced in the stratum radiatum of CA1 by Schaffer collateral stimuli at 50 ms interval as the ratio of the amplitude of the second field potential to the first one. As shown in Fig. 4a,b, PPR values showed a decline along the hippocampal septo-temporal axis (F = 70.313; p < 0.001; Post-hoc comparisons: DH vs IH, p < 0.001, t = 6.757; DH vs VH, p < 0.001 t = 11.437; IH vs VH, p < 0.001; t = 3.620), supporting previous findings reported for the VH and DH rat regions^12^,^15^. The variation of PPR indicates that in VH glutamate release probability at Schaffer collateral synapses is remarkably higher in VH compared to DH.

We next evaluated long-term plasticity (LTP) at Schaffer collateral synapses by measuring changes in fEPSP amplitude with time after high frequency stimulation (HFS, 2 trains of 100 stimuli at 10 ms interval, separated by 3 s). Figure 4c shows that mean values of fEPSP slope were persistently increased in slices prepared from the DH, IH and VH. The amplitude of LTP, measured at 35 min after HFS, declined gradually in slices prepared from DH to VH (F = 6.90, p = 0.005; Post –hoc comparisons: DH vs VH, p = 0.004, t = 3.70; IH vs VH, p = 0.164, t = 1.44; IH vs DH, p = 0.19, t = 1.70), similar to reports in rats^14^,^16^. Thus, both short- and long-term-plasticity at synapses that excite CA1 pyramidal cells vary in a similar way in slices taken from different sites along the septo-temporal axis.

### Stochastic and evoked GABA release differences in dorsal and ventral hippocampus

**Figure 5:**
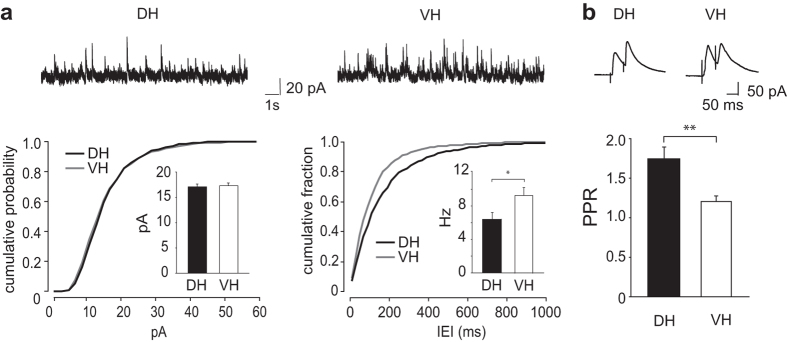
Differences in release from inhibitory synapses in slices of DH and VH. Whole cell records from CA1 pyramidal cells. (**a**) Histograms of mean mIPSC amplitude (left) and frequency (right) in DH and VH. Above: representative patch-clamp recordings of mIPSCs in CA1 pyramidal neurons in DH and VH. Bottom: cumulative probability histogram of mIPSCs amplitude (left) and IEI (right) from DH (black line) and VH (gray line) cells. Mean amplitude for mIPSCs: DH, 16.3 ± 0.7 pA (n = 10/8/8, Total number on events, 2010); VH, 16.6 ± 0.6 pA (n = 13/10/10, Total number on events, 2610). Mean frequency for mIPSCs: DH: 6.4 ± 0.8 Hz, (n = 10/8/8); VH: 9.3 ± 0.9 Hz (n = 13/10/10). Note the shift toward the left in IEI curves indicating an increase in mIPSCs frequency of VH cells. (**b**) Mean PPR for evoked IPSCs in CA1 pyramidal cells of DH and VH. Mean PPR: DH, 1.75 ± 0.15, n = 16/10/10; VH, 1.19 ± 0.07, n = 16/11/11). Above: evoked IPSCs showing differences in PPR at inhibitory synapses of DH and VH. Data shown as mean ± S.E.M. *Indicates p < 0.05 and **indicates p < 0.01. Traces are an average of the ten consecutive slices.

Does a septo-temporal gradient also exist for inhibitory synaptic function? We compared for the first time in rodents miniature action potential-independent IPSCs (mIPSCs, recorded in the presence of TTX, 0.5 μM) from CA1 pyramidal neurons in slices prepared from DH and VH. Inhibitory synaptic events were isolated in whole-cell recordings at a holding potential of 0 mV near the reversal potential of glutamatergic synaptic events. Figure 5a shows that there was no difference in mIPSC amplitude between VH and DH (F = 0.116, p = 0.73). Indeed, the cumulative distribution plots for amplitudes were similar. In contrast mIPSC frequency in CA1 pyramidal cells of DH slices was significantly lower than those of VH regions (t = 2.330; p = 0.03). Accordingly, the inter-event intervals (IEI) cumulative probability plot of VH cells was shifted toward the left (Fig. 5a).

As for excitatory events at Schaffer collateral synapses, we tested the PPR for GABAergic IPSCs induced by focal stimuli in stratum radiatum at an interval of 50 ms. As shown in Fig. 5b, the PPR was higher for inhibitory synapses formed with CA1 pyramidal cells in slices from DH regions (t = 3.332 p = 0.002). Thus both mIPSCs and PPR data suggest that, similarly to glutamate release (Fig. 4a), GABA release may be higher in slices from VH rather than DH.

## Discussion

This study demonstrates consistent, significant changes along the longitudinal, septo-temporal axis both in intrinsic neuronal properties of CA1 pyramidal cells and in the features of synapses that excite and inhibit them. To perform this analysis, we collected and straightened the hippocampus in order to apply a coordinate system that allowed to identify the longitudinal position of each transverse slice. We found that pyramidal cells of DH were less excitable than those of IH or VH, the short- and long-term plasticity were systematically reduced from the DH to the VH and GABAergic transmission was higher in VH than in DH, indicating an increased probability of both GABA and glutamate release in the VH compared to more dorsal regions of the hippocampus.

### Intrinsic excitability and sub-threshold membrane properties

We found that the excitability of CA1 pyramidal neurons was not homogenous along the longitudinal hippocampal axis. Whole cell recordings revealed a more depolarized RMP and a higher Rin for pyramidal cells in VH than in DH slices. Differences in both membrane conductances and neuronal morphology may contribute to the observed differences. Indeed, in rats, membrane conductances near rest differ from DH to VH (i.e. Kv7^23^, HNCN^24^ and GIRK^25^) and dendritic surface area of DH neurons is larger than that of VH ones^10^,^11^,^26^. We did not observe any significant difference in the average membrane capacitance between VH and DH cells but we measured a similar specific conductance, suggesting that differences in RMP and Rin might depend on both cell dimension and ionic conductances. In addition, we found that a little current injection (50 pA) was ineffective in DH but evoked spikes in VH neurons indicating that ventral neurons are more excitable. This finding, corroborated by the results on population spike obtained by local field potential analysis (that do not perturb the intracellular ionic milieu), is in line with previous studies performed in the rat^10^,^11^.

### Synaptic plasticity along hippocampal septo-temporal axis

According to the input–output relationship, basal synaptic function did not differ between VH and DH. However, we found differences in both short-term and long term plasticity of excitatory synapses made by Schaffer collaterals onto CA1 neurons along the dorsal-ventral axis. In particular, paired pulse facilitation, a short-term synaptic plasticity, was reduced from dorsal to ventral regions. Since it depends on a transient increase of transmitter release due to residual presynaptic calcium, PPR differences indicate that glutamate release probability is higher in VH compared to DH^12^,^15^. Our results could both rely on a gradual change along the axis, or be due to a segregated regulatory mechanisms in the two poles.

Our data showed a lower degree of LTP at Schaffer collateral synapses in VH than in DH^12^,^13^,^14^,^16^,^19^, with intermediate levels in IH. Differences in LTP are possibly related to structural factors, events during HFS or inhibitory control. Structural factors may include a greater total dendritic surface in pyramidal cells in DH than those in VH^10^,^12^,^14^,^15^,^16^. Differences in inhibitory control of responses to tetanic stimuli^27^,^28^, in metabotropic glutamate receptor-linked release of calcium from intracellular store^14^ or in NMDA receptor expression from dorsal to ventral portion^1^,^27^,^28^, might also contribute to lower levels of long-term plasticity in VH. Differences in the strength of systems that modulate transmitter release could also be involved in the variation of LTP extent. For instance, aminergic and peptidergic innervation is higher in VH, while the expression of adenosine A1 and muscarinic receptors is lower^1^. A lower density of pre-synaptic A1 receptors in VH might also contribute to different levels of short-term PPR.

### Stochastic and evoked GABA release differences in dorsal and ventral hippocampus

While neurochemical differences along the septo-temporal axis have been described for several neurotransmitters^1^, little data is available for the GABAergic system. These limited results provide an indirect evidence that inhibition has variable effects on intrinsic network excitability. For instance, field records in rats suggest that paired-pulse inhibition of population spikes, presumably under inhibitory control, is weaker in slices from VH than DH^28^,^29^. In addition, differences in GABA_B_ receptor subtypes may underlie a different control of network excitability in DH and VH. Finally, disinhibition due to GABA_A_ receptor blockade induces more prominent synchronous field potentials in VH than in DH^30^. In order to directly assess GABAergic neurotransmission, we recorded for the first time, to the best of our knowledge, whether spontaneous miniature IPSCs and action potential-driven GABAergic responses vary along the hippocampal longitudinal axis. Differences in both mIPSC frequency and PPR suggest that GABA release probability is higher in VH than in DH. Such differences in neurotransmitter release probability could depend on terminal size, presynaptic calcium channels, autoreceptors or other components of the synaptic release machinery. In addition, the distribution of subclasses of interneurons impinging onto pyramidal cells may contribute to the differences in GABA release along the axis. Indeed, although some discrepancies have been reported^27^,^31^,^32^,^33^, both mice and rats show a dorso-ventral distribution of various types of interneurons. However, since we found that glutamate has a similar release probability gradient of GABA, we speculate that the same regulatory mechanisms may control the release of both neurotransmitters. This mechanism may involve neuromodulators, including acetylcholine and serotonin, which regulate the release of both GABA and glutamate^34^,^35^. These neuromodulators are diffusely released, act by volume transmission and make stronger projections to ventral hippocampus than to dorsal regions^1^. These topographically organized projections may increase basal levels of both GABAergic and glutamatergic release in ventral regions, in line with our results. Further elucidation of the potential regulating mechanisms and the functional role of differential transmitter release probability is warranted.

In conclusion, our findings illustrate hippocampal organization in the mouse, the most commonly used species in bio-medicine laboratories worldwide. In particular, we described the electrophysiological properties of CA1 neurons at different dorso-ventral sites, contributing to the understanding of segregation of the hippocampal function along its septo-temporal axis. In addition, the present results reveal that the mouse share with the rat most of the hippocampal organization which partially overlaps also that of the human^1^, confirming that the hippocampal structure is phylogenetically conserved.

## Methods

### Animals

All experiments were conducted in conformity with European Directive 2010/63/EU and the Italian D.lg. 4.05.2014 and all methods were carried out in accordance with relevant guidelines and regulations. Adult (8–12 weeks old) male C57BL6 mice (26), used for the experiments, were housed under a 12 h light/dark cycle at a temperature of 21 ± 1 °C with food and water provided ad libitum. All efforts were made to minimize the number of animals used and their suffering. The experimental protocol (N. 134/2015-PR) was approved by the Italian Ministry of Health.

### Immunofluorescence

Immunofluorescence experiments to visualize neuronal nuclei with the Hoechst dye were performed as previously described^36^. Briefly, coronal cryostat hippocampal sections (10 μm) were incubated with 3% goat serum in 0.3% Triton X-100 for 1 h at RT before exposure to the Hoechst dye. Fluorescent images were acquired with a CoolSNAP camera (Photometrics) coupled to an ECLIPSE Ti-S microscope (Nikon). Successive slices were scanned at x10 to obtain a single image per section. All coronal sections from a hippocampus were analyzed using MetaMorph 7.6.5.0 software (Molecular Device).

### Hippocampal slice preparation

Animals were anesthetized with halothane and then decapitated. The whole brain was rapidly removed and immersed in ice-cold artificial cerebrospinal fluid (ACSF) solution containing (in mM): NaCl 125, KCl 4.4, CaCl_2_ 2.5, MgSO_4_ 1.5, NaHPO_4_ 1, NaHCO_3_ 26 and glucose 10 which was oxygenated with 95% O_2_, 5% CO_2_ to maintain a pH close to 7.4. Hippocampi were rapidly removed from the brain and elongated (Fig. 1a). The dorsal (or septal) hippocampus was identified by its proximity to the retrosplenial cortex and the ventral (or temporal) was close to the amygdaloid complex. Isolated hippocampi were placed in an Agar block (2.25% weight/volume in bi-distilled water) and fixed on a plate (Fig. 1b) for slicing with a vibratome (Thermo Scientific, USA). Transverse 350 μm thick slices were cut at 4 °C from the entire length of each hippocampus except the extremities of length about 250 μm. The longitudinal position of each slice was identified as distance, in μm, from the dorsal pole before recording. Results are presented with septo-temporal recording sites schematically divided into DH, IH and VH regions of 2 mm each (Fig. 1c). After preparation, slices recovered for 1 h at 30 °C in a chamber containing oxygenated ACSF. Importantly, the slice isolation procedure seems not preclude functional differentiation along the septo-temporal axis.

### Electrophysiology

#### Extracellular field recordings

For field recordings, individual slices were transferred to an interface slice-recording chamber (BSC1, Scientific System Design Inc) with a total fluid dead space of about 3 ml where they were visualized with a Wild M3B stereomicroscope (Heerbrugg, Switzerland). They were maintained at 30–32 °C and superfused with ACSF at 2.5 ml/min by a peristaltic pump. A concentric bipolar stimulating electrode (SNE-100 × 50 mm long, Elektronik–Harvard Apparatus GmbH) was placed at a constant distance in the stratum radiatum to stimulate Schaffer collateral fibres in the CA1 region. Stimuli consisted of 100 μs constant current pulses of variable intensity, applied at 0.05 Hz. An ACSF-filled glass micropipette (0.5–1 MΩ) was placed at 200–600 μm from the stimulating electrode to measure orthodromically-evoked field extracellular postsynaptic potentials (fEPSP). They were recorded and filtered (low pass at 1 kHz) with an Axopatch 200 A amplifier (Axon Instruments, CA) and digitized at 10 kHz with an A/D converter (Digidata 1322 A, Axon Instruments). Stimulus intensity was adjusted to evoke fEPSPs of amplitude about 50% of maximal amplitude with minimal contamination by a population spike. Baseline evoked responses were monitored online over 10 min and only slices with stable fEPSP amplitudes were included and fEPSP values were averaged over 1 min (n = 3). Input–output (I–O) relations for field potentials were measured at the start of each experiment by applying a series of stimuli of increasing intensity to the Schaffer collaterals. Population spikes initiated at higher stimulus intensities were apparent as an opposing deflection superimposed on the fEPSP, identified by visual discrimination. Population spike occurrence was detected as a change of the field slope from monophasic to biphasic shape. The threshold intensity needed to induce a population spike was calculated by dividing the effective stimulus intensity (V) by the maximal stimulation intensity which induces fEPSPs of maximal initial slope. The paired-pulse ratio (PPR) was measured from responses to two synaptic stimuli at 50 ms inter-stimulus interval. The PPR was calculated as the ratio between the fEPSP amplitude evoked by the second stimulus (A2) divided by that induced by the first (A1; A2/A1). Since PPR may vary with stimulus intensity, especially for small or large fEPSPs, we adjusted stimuli to evoke initial fEPSPs of 40 ± 60% of maximal amplitude determined from input-output curves. The stimulus protocol to induce LTP consisted of 2 trains of stimuli at 100 Hz, of 1 s duration and separated by 3 s. Averaged fEPSPs at 35 min after these trains were normalized to baseline values before high-frequency stimulation.

#### Patch clamp recordings

Slices were visualized using a Leica DM LFS microscope. Whole-cell patch clamp recordings were made from CA1 pyramidal neurons, typically in the middle of the pyramidal layer at room temperature using a Multiclamp 700B amplifier (Molecular Devices, USA). ACSF was perfused at a rate of approximately 2 ml/min using a gravity-driven perfusion system. Cell capacitance and access resistance were monitored and records were stopped if either parameter changed by more than 20%. Glass pipettes (3–4 MΩ) were pulled with a vertical puller (PC-10, Narishige). For voltage clamp records they were filled with 140 mM Cs Methanesulfonate, 10 mM Hepes, 0.5 mM EGTA, 2 mM Mg-ATP, 0.3 mM Na3-GTP and 2 mM MgCl_2_ (295–300 mOsm, pH 7.2). For current clamp records pipettes were filled with 120 mM Kgluconate, 20 mM KCl, 10 mM Hepes, 4 mM Mg-ATP, 0.3 mM Na3-GTP and 4 mM NaCl (280–290 mOsm, pH 7.2). Signals were acquired with a DigiData-1440A digitizer controlled by pCLAMP-v10 software (Molecular Devices, USA). They were sampled at 10 kHz and low-pass filtered at 2 kHz.

Resting Membrane Potential (RMP) was measured as the voltage with no injected current. All voltages are corrected for a calculated liquid junction potential of 12 mV. Membrane capacitance was estimated as the total charge (i.e., the current integral, Qstep) mobilized in each cell by a 10 mV hyperpolarizing step (Vstep): Qstep/Vstep. The specific conductivity of the membrane was estimated as the ratio between conductance (G = 1/Rin) and capacitance (C, nS/μF). The somatic Rinput was measured from responses at resting potential to step current injections of amplitude −50 pA to 50 pA in 10 pA increments and 800 ms duration. Relations between firing frequency and injected current were examined by measuring action potentials elicited by somatic current injections from 50 to 350 pA starting from RMP.

GABAergic membrane currents were recorded from pyramidal cells clamped at 0 mV. At this potential, close to the reversal of excitatory currents, the Cl^−^-mediated outward inhibitory currents were outward, given the estimated reversal potential near to −80 mV. The validity of this approach is supported by the observation that 100 μM picrotoxin completely eliminated all spontaneous outward current activity recorded at 0 mV (not shown).

Miniature IPSCs were recorded in the presence of TTX (0.5 μM, Tocris Bioscience, Bristol, United Kingdom) perfused after an initial 10 min baseline period.

The PPR of IPSCs was measured from events elicited with a concentric bipolar stimulating electrode (SNE-100 × 50 mm long Elektronik-Harvard Apparatus GmbH, Crisel Instruments, Rome, Italy) placed in the stratum radiatum. Pairs of stimuli (ISI 50 ms) were applied every 20 sec and delivered through a A320R Isostim Stimulator/Isolator (WPI) (intensity range, 0.2–0.4 mA). The PPR was calculated by measuring the amplitude of each IPSC relative to a 2 ms baseline period starting 3 ms before stimulation. IPSCs were recorded at 20 kHz and filtered with a low-pass Bessel filter at 4 kHz.

### Data analysis

Data were stored on a computer using pClamp 9 software (Axon Instruments) and analyzed off-line with Clamp-fit 9 program (Axon Instruments). Miniature IPSCs were analyzed using MiniAnalysis software (Mini Analysis, Synaptosoft Fort Lee, NJ, USA) with detection threshold set at 5 pA. For cumulative probability plot comparisons we used Kolmogorov-Smirnov test (Mini Analysis, Synaptosoft Fort Lee, NJ, USA). For field recordings n/N refers to numbers of slices/mice. For patch clamp recordings n/N/N1 refers to numbers of cells/slices/mice. All parameters are reported as mean ± SEM and tests of significance were made with the *t* test (p < 0.05 was significant) or with the ONE-way ANOVAs with a two-sided alpha of 0.05 and a power of 0.80. Post hoc comparisons were performed using the Holm–Sidak’s test. Only for comparison of the firing frequency at 50 pA in the F-I curve (were normality test failed) the Mann-Whitney rank Sum test were used.

## Additional Information

**How to cite this article**: Milior, G. *et al*. Electrophysiological Properties of CA1 Pyramidal Neurons along the Longitudinal Axis of the Mouse Hippocampus. *Sci. Rep.* **6**, 38242; doi: 10.1038/srep38242 (2016).

**Publisher's note:** Springer Nature remains neutral with regard to jurisdictional claims in published maps and institutional affiliations.
